# Computer Aided Design Modelling and Finite Element Analysis of Premolar Proximal Cavities Restored with Resin Composites

**DOI:** 10.3390/ma14092366

**Published:** 2021-05-01

**Authors:** Amanda Guedes Nogueira Matuda, Marcos Paulo Motta Silveira, Guilherme Schmitt de Andrade, Amanda Maria de Oliveira Dal Piva, João Paulo Mendes Tribst, Alexandre Luiz Souto Borges, Luca Testarelli, Gabriella Mosca, Pietro Ausiello

**Affiliations:** 1 Department of Dental Materials and Prosthodontics, Institute of Science and Technology, São Paulo State University (UNESP), São José dos Campos, SP 12245-000, Brazil; amanda.matuda@unesp.br (A.G.N.M.); marcos.motta@unesp.br (M.P.M.S.); guisdandrade@hotmail.com (G.S.d.A.); amodalpiva@gmail.com (A.M.d.O.D.P.); alexanborges@gmail.com (A.L.S.B.); 2 Department of Dentistry, University of Taubaté (UNITAU), Taubaté, SP 12020-270, Brazil; joao.tribst@gmail.com; 3 Department of Oral and Maxillo Facial Sciences, “Sapienza” University of Rome, 00161 Rome, Italy; luca.testarelli@uniroma1.it; 4 Department of Neurosciences, Reproductive and Odontostomatological Sciences, School of Dentistry, University of Naples Federico II, 80138 Naples, Italy; gabriellamoscaf@gmail.com

**Keywords:** mechanical stress, finite element analysis, dental inlays, dental prosthesis, dental materials

## Abstract

This study evaluated the stress distribution in five different class II cavities of premolar models restored with conventional or bulk-fill flowable composite by means of finite element analysis (FEA) under shrinkage and occlusal loading. An upper validated premolar model was imported in the software, and five class II cavities with different occlusal extensions and dimensions were prepared: horizontal cavity on the mesial surface (horizontal slot), mesio-occlusal cavity, mesial cavity (vertical slot), tunnel type cavity and direct access cavity. The models were restored with conventional or bulk-fill flowable resin composite. The tested materials were considered as homogeneous, linear, and isotropic. The Maximum Principal Stress criteria was chosen to evaluate the tensile stress results. The lowest shrinkage stress value was observed in the direct access cavity restored with bulk-fill flowable resin composite (36.12 MPa). The same cavity, restored with conventional composite showed a score of 36.14 MPa. The horizontal slot cavity with bulk-fill flowable showed a score of 46.71 MPa. The mesio-occlusal cavity with bulk-fill flowable had a score of 53.10 MPa, while with conventional composite this was 55.35 MPa. Higher shrinkage stress was found in the vertical slot cavity with conventional resin 56.14 MPa, followed by the same cavity with bulk-fill flowable 56.08 MPa. Results indicated that the use of bulk-fill flowable composite resin more significantly decreased the polymerization shrinkage stress magnitude. The larger the cavity and the volume of material necessary to restore the tooth, the greater the residual stress on enamel and dentin tissue.

## 1. Introduction

Caries is a common oral disease caused by initial demineralization of the tooth structure because of bacteria acid production in the presence of fermentable carbohydrates, and following enamel and dentin mechanical resistance loss [1]. The tooth decay can emerge in different forms, from a small, demineralized area in the enamel to a large cavity in the dentin with or without pulpal involvement [2]. In some cases, when proximal caries (class II) progress extensively in dentin, it is necessary to remove the intact marginal ridge to gain access to the carious lesion [1].

Currently, it is suggested the cavity design should be indicated exclusively by the caries extension, since nowadays the composite resin bond strength with the dental tissues allows a more conservative approach [3]. The principal advantage of this minimally invasive dentistry is to preserve the healthy dental structure as much as possible. Thus, it is reported that the survival of restorations decreases as the size and complexity of the cavities increase [4]. Class II preparations with marginal ridge involvement can reduce the tooth resistance [5]. There are alternative designs of class II cavities and approaches, such as tunnel restoration, that can increase the final integrity and resistance of the teeth, preserving the marginal ridge [6].

For these class II cavity models, the use of resin composites as a restorative material is the first option due to its ability to mimic the missing dental tissues, easy handling, proper adhesion, adequate mechanical resistance and mechanical behavior of enamel and dentin [7]. In order to attenuate inherent problems of resin composite restoration such as the polymerization shrinkage and the use of the several increments technique, the use of bulk-fill resin composite has been suggested because of lower shrinkage stress. Despite the proposed use of a composite single increment to restore 4 mm deep dental cavities, to optimize clinical time in small or medium size cavities, the clinical benefits of an extensive bulk-fill composite application requires more study [8]. It is known that the bulk-fill flowable resin composite has advantages in terms of shrinkage stress distribution versus the resin incremental filling technique, such as lower rate of polymerization shrinkage [9,10].

In order to study some complex problems, the finite element method can be used for quantitative and qualitative analysis of the mechanical behavior of dental structures [11,12]. Thus, the objective of this study was to evaluate the stress under vertical occlusal loading and polymerization shrinkage stress in five different models of class II cavities adhesively restored with conventional or bulk-fill flowable resin composites by means of finite element analysis (FEA).

The null hypothesis was (1): There would be no difference in the proximal cavities’ stress concentration with the application of a bulk or conventional resin composite. (2): The different cavities’ design would not exhibit similar stress magnitudes.

## 2. Materials and Methods

A first upper premolar model, previously validated [12], was imported into the computer aided design (CAD) software (version 4.0 SR8, McNeel North America, Seattle, WA, USA) as a standard model for all simulations. Different parts of the tooth, including pulp, dentin, enamel, periodontal ligament, cancellous bone and cortical bone were designed in this present study.

**Figure 1:**
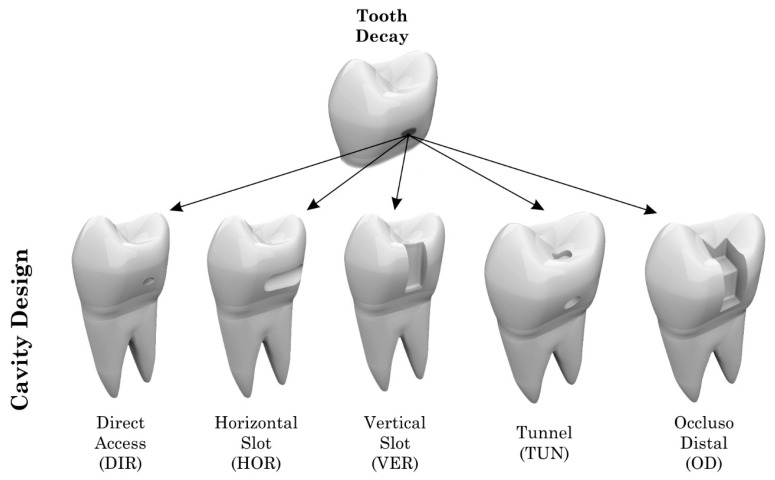
Different models of class II cavities evaluated in this study.

Different models of class II cavities with specific extensions, dimensions and final volumes (Table 1) were prepared in the teeth in the five groups of class II cavities based on the clinical approach used to restore proximal tooth decay: horizontal cavity on the mesial surface (horizontal slot), mesio-occlusal cavity, mesial cavity (vertical slot), tunnel type cavity or direct access cavity (preparation restricted to caries injury). The cavities were restored with conventional composite resin (Filtek Z350, 3M-Espe, Sumaré, Brazil) or bulk-fill flowable (Filtek Bulk Fill, 3M-Espe, Sumaré, Brazil) [13], except for the tunnel preparation, which was indicated as having received only the bulk-fill flowable (Figure 1).

**Figure 2:**
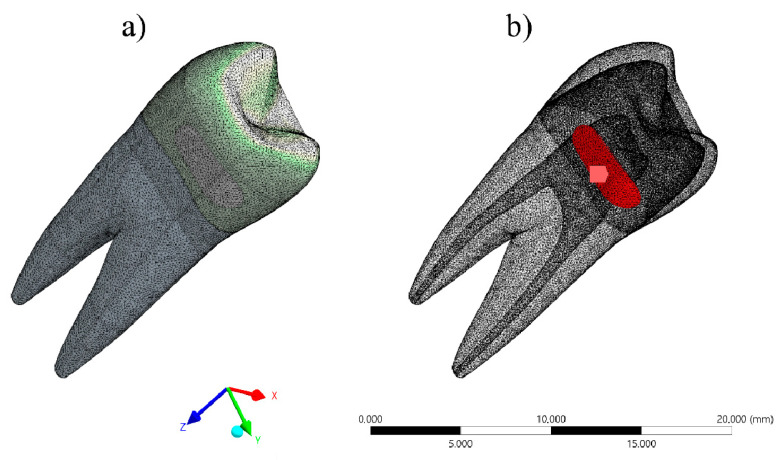
(**a**) Mathematical model during meshing process and (**b**) horizontal slot model with composite resin polymerization shrinkage as loading.

The CAD models were exported as STEP files to the analysis software (ANSYS 17.2, ANSYS Inc., Houston, TX, USA). The mesh was created with tetrahedral elements, and the contacts were considered connected (Figure 2). The properties of each material were loaded and the models were considered linear, isotropic and homogeneous.

Polymerization shrinkage was simulated by thermal analogy [14]. The temperature was reduced by 1 °C, and the linear shrinkage value (post gel shrinkage) was simulated based on the coefficient of linear thermal expansion (Figure 2).

**Figure 3:**
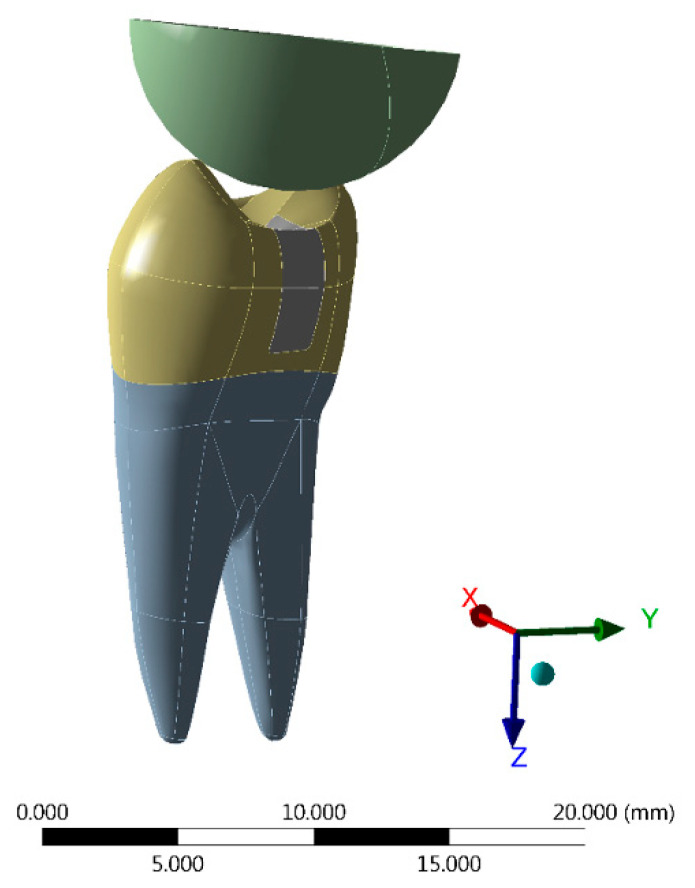
Loading method performed in the present simulation using a stainless steel spherical loading device.

For the models restored with conventional resins, two increments were simulated. For this, the stress was generated in two steps (one for each portion of the resin composite) [15]. A static mechanical structural analysis was used to calculate the stress distribution after application of a 100 N occlusal load simultaneously [16]. The occlusal contact points were defined at the tips of the cusps using a stainless steel spherical loading device model [16] (Figure 3). The Von Mises criteria was used to observe the coherence of the numerical simulations. The Maximum Principal Stress criteria was chosen to evaluate the tensile stress results. All materials were considered homogeneous, linear, and isotropic, and their mechanical properties are summarized in Table 2.

## 3. Results

**Figure 4:**
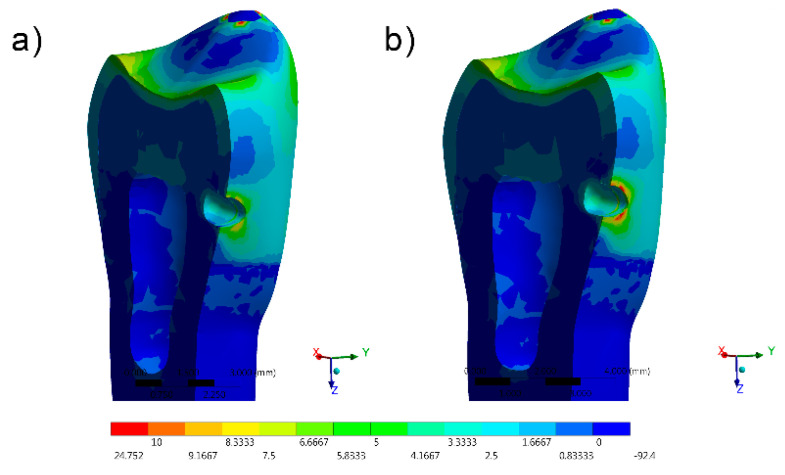
FEA Maximum Principal Stress results in the direct access design. (**a**) Bulk-fill flowable and (**b**) Conventional.Figure 5 FEA Maximum Principal Stress results in the mesio-occlusal design. (**a**) Bulk-fill flowable and (**b**) conventional.

**Figure 5:**
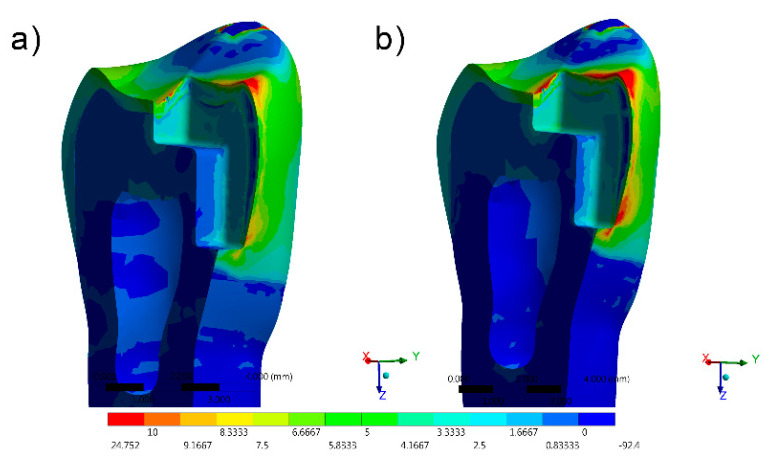
FEA Maximum Principal Stress results in the mesio-occlusal design. (**a**) Bulk-fill flowable and (**b**) conventional.

**Figure 6:**
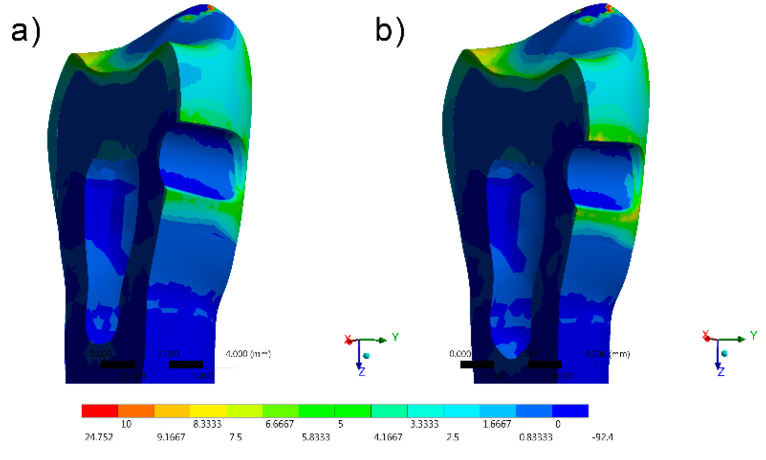
FEA Maximum Principal Stress results in the horizontal slot design. (**a**) Bulk-fill flowable and (**b**) conventional.

**Figure 7:**
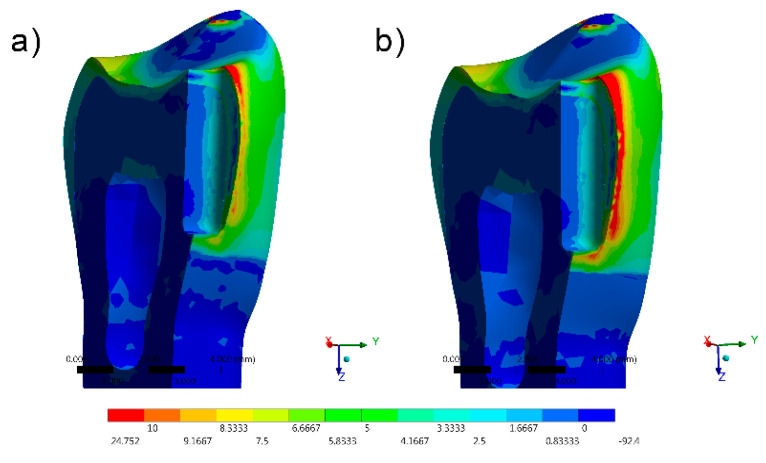
FEA Maximum Principal Stress results in the vertical slot design. (**a**) Bulk-fill flowable and (**b**) conventional.

**Figure 8:**
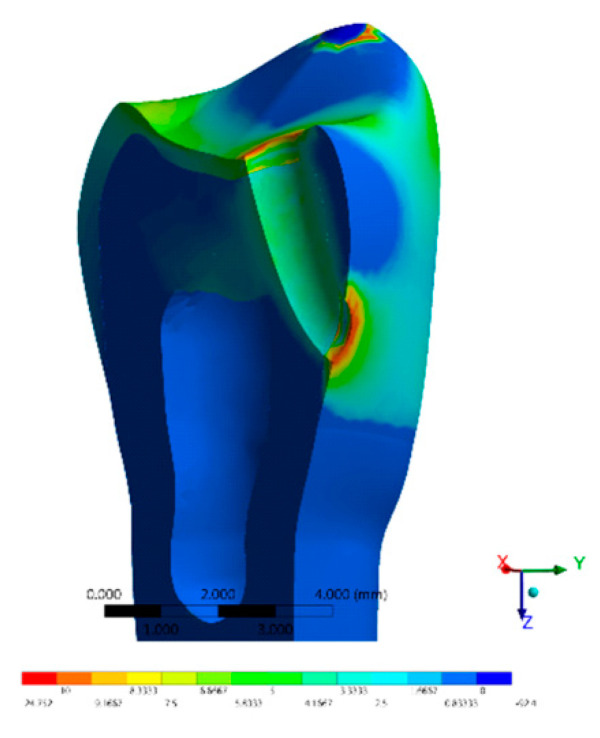
FEA Maximum Principal Stress results in the tunnel design with bulk-fill flowable resin.

The stress distribution results for all groups are shown as colorimetric graphs in Figure 4, Figure 5, Figure 6, Figure 7 and Figure 8. Different colorimetric fringes represent different stress magnitudes, visible in the horizontal scale. Hot colors mean higher tensile stress magnitude, while cold colors indicate lower tensile stress magnitude. In general, the composite resin factor and cavity geometry affected the stress distribution in the tooth. Regardless the cavity design, the restorations with conventional composite resulted in a higher stress concentration.

The stress peaks were recorded in two different moments; first, only the polymerization shrinkage stress effect was present as resultant from the resinous material applied to fill the cavity (Table 3). In the second moment, the resultant stress was calculated after the loads vertically applied on the cusps, simultaneously in combination with shrinkage effects (Table 4).

The resultant stress distributions for the models were compared and examined, and the Maximum Principal Stress was designated as analysis criteria. Regardless of the cavity design, the highest stress concentration in the enamel tissue occurred in the cavosurface angle at the restoration margin, except in the vertical slot design. In addition, the residual stress during the resin composite polymerization shrinkage was reflected in models with proportional stress magnitude after the compressive loading application.

The most favorable result, with the lowest shrinkage stress, was observed in the direct access cavity before compressive loading (10.09 MPa) and after compressive loading (36.12 MPa) The same cavity, restored with conventional resin, presented a slightly greater (11.13 and 36.14 MPa before and after loading) magnitude of shrinkage stress.

Favorable results were also found in the restoration of the horizontal slot cavity, where the bulk-fill flowable was able to reduce the stress in comparisons with the conventional resin composite; however, the stress data were higher than the horizontal slot. Tunnel cavity restorations with bulk-fill showed intermediate results.

In comparison with the direct access and horizontal slots, higher stress values were observed in the mesio-occlusal cavity design restoration for the bulk-fill flowable and conventional resin composites.

The results most prone to failure were calculated when restoring the vertical slot cavity with conventional resin, followed by restoring the same cavity with bulk-fill flowable. In this case, it was observed that the whole system was compromised, even when the bulk-fill flowable was used.

## 4. Discussion

The null hypothesis was that there would be no difference in the proximal cavities restored with the application of a bulk or conventional resin composite. The results showed that different cavity design exhibited different magnitudes, and could affect the dental treatment biomechanics. Thus, both the hypotheses were rejected.

The bond strength among restorative materials and dental tissues must be effective at the interface of the restored cavity, mimicking the mechanical properties of lost mineralized tissues and limiting marginal leakage and cusp deflection [19,20]. Morphology, function and esthetics must also be replicated [17].

The results of a literature review suggest that composite resin restorations can demonstrate clinically acceptable results in a long-term period of more than 15 years of follow-up [21]. According to the literature [22] the annual failure rate of direct composite resin restorations is 2.3% in class II cavities. One of the main causes is the shrinkage stress, which has been widely investigated in previous studies [14,23,24,25,26] and considered a significant factor in the clinical failure of direct adhesive restorations. According to the literature, cavity geometry, material properties and restoration techniques are some of the factors considered significant to the increase of polymerization shrinkage stresses [26,27,28].

The bond strength can decrease at the tooth-restoration interface or at the margins of the restoration, resulting in marginal stains, internal and marginal fissures, micro-cracks, secondary caries and premature restoration loss [29,30]. Restoration failure is consequently associated with a high magnitude of residual polymerization shrinkage stress [29,30,31,32,33,34,35,36,37,38,39,40].

According to the obtained results, cavities adhesively restored with conventional composite resin presented higher residual stress concentrations. Therefore, this result is in agreement with the literature which has reported that high modulus conventional resin composite can promote a significant effect on the marginal microleakage as a shrinkage, debonding or fracturing of the adhesive interface [15,34,35].

The bulk-fill flowable resin composites showed in this investigation less polymerization shrinkage stress because of the described presence of modulators and polymerization monomers capable to relieve stress [31,32,33,34,35,36,37,38,39,40,41]. The results of the present study corroborate previous theoretical findings, which conclude that the use of bulk-fill resins can contribute to reducing the undesirable effects of polymerization shrinkage [15,23].

For this study, different types of class II preparations were selected, with the aim of simulating the restorative procedures performed routinely in different patients. Direct access is performed mainly in cases of local carious lesions and with a reduced extension, removing only the necessary tissue with no needs for additional retentive forms. The preparation of the mesial cavity (vertical slot) occurs when there is involvement of the area affected by caries in the proximal region, with access to the rupture of the marginal ridge. In this case, the preparation is self-retaining because the buccal and lingual walls converge towards each other to the occlusal surface. Compared to the vertical slot, the preparation of a horizontal slot is more conservative because it is strictly proximal and can be achieved by buccal or lingual, preserving the occlusal surface and the marginal ridge. The mesio-occlusal cavity, on the other hand, is performed when the carious lesion compromises two sides of the dental structure, with the rupture of the marginal ridge and the occlusal surface. It is a more extensive cavity with a limited c-factor, and in this cavity design, the walls of the proximal box converge towards to the occlusal. In incipient caries located on the proximal surfaces of posterior teeth and with marginal ridges free of lesions, tunnel-type preparations can be performed where the marginal ridge is preserved, and access is granted through the occlusal surface [39].

In a previous study [13] that aimed to evaluate the effect of extension and type of class II cavity on the biomechanical properties of teeth, the authors concluded that bigger mesio-distal II cavities, as well as lower amounts of the mesial and distal remaining walls, increased the stress concentrations in the enamel [13]. This is according to the results obtained in this present study, where the larger preparations presented greater stress magnitude. Regarding the direct access preparation design, there are no studies in the literature that have evaluated it.

In relation to the types of cavities, larger restorations promote a greater increase in stress at the tooth-restoration interface [10]. According to previous studies [21,22,23,24] the use of resin-based bulk filling materials is not recommended for large MOD (mesio-occluso-distal) Class II adhesive restorations. Mechanical behavior under loading and shrinkage stress is monitored by several factors including the enamel and dentine volumes lost. In this present study, however, the analyzed class II cavities are limited to small–medium size volumes. Here the use of bulk-fill material was respective of not only the limited depth of the cavity, but mainly the influence of the c-factor and of the total refilling volumes.

The preparation of a tunnel-type cavity can be considered more difficult compared to the preparation of the other types of class II cavities [39]. It is reported that one of the main advantages of this type of preparation is the possibility of keeping the marginal ridge intact; however, there may be failures due to the fracture of the marginal ridge that continues to occur even after 24 months after completion of the restoration [31].

According to the data obtained in previous reports [40] and the results obtained in this study, the class II tunnel type design does not present any mechanical advantage to decrease the calculated stress magnitude. Due to the technical difficulties in performing this type of preparation, the horizontal slot or the direct access design should be preferred for the operative treatment of medium-sized class II proximal cavities [31].

Some intrinsic limits of the current finite element analysis involve the use of a model with an isotropic resin composite, without defect populations in the material, perfectly bonded in all cavities and with a linear behavior. Despite all the advantages of FEA [42,43,44], it must be taken into account that the precision of its results also has tolerance limits, which must be considered during the results interpretation [40,41,42,43,44].

This present study showed that the use of bulk-fill flowable resins could reduce the residual stress of small proximal cavities. In addition, class II preparations of the mesio-occlusal type and vertical slot, due to the size and geometry of the cavity, may present an increase in shrinkage stress and, consequently, greater stress at the tooth-restoration interface.

Despite the results reported with the present in silico evaluation, some limitations should be considered when interpreting them. The present analysis did not consider the pH variation simulation, cyclic loading, restoration adhesive failure, biofilm formation, temperature variation or different antagonists. In addition, the simulated materials were not orthotropic or anisotropic materials, and the obtained results were not calculated considering data deviation or systematic error that can be present in biological tissues and complex systems. However, further fatigue lifetime studies should be developed, considering the S–N curve, to provide a more complete in vitro evaluation prior to clinical trials using prospective studies.

## 5. Conclusions

Based on this information and despite the limitations of this study, it can be concluded that:

- Regardless the cavity design, the use of bulk-fill flowable resin composite is a viable option to restore the missing dental tissue.
- Smaller class II cavities can be conveniently restored with low shrinkage resin composites to reduce the residual stress and the adhesive failure risk.
- Conservative preparations should be performed in order to reduce the volume of resin composite material, and the stress of the direct proximal restorations.

## Figures and Tables

**Table 1:** Design inputs for each different type of proximal cavity simulated in the present study.

Model	Height	Width	Depth	Volume
Mesio-Occlusal	4.55 mm	2.19 mm	3.42 mm	19.20 mm^3^
Direct Access	0.94 mm	1.47 mm	1.34 mm	0.87 mm^3^
Vertical Slot	4.93 mm	2.0 mm	1.36 mm	9.78 mm^3^
Horizontal Slot	2.14 mm	5.45 mm	1.22 mm	5.44 mm^3^
Tunnel	4.25 mm	1.49 mm	3.30 mm	6.50 mm^3^

**Table 2:** Mechanical properties of the materials used in the tests.

Structure	Elastic Modulus (GPa)	Poisson’s Ratio	Coefficient of Thermal Expansion	Composition	Reference
Enamel	80.0	0.30	-	-	[17]
Dentin	18.0	0.23	-	-	[17]
Pulp	0.000003	0.45	-	-	[18]
Ligament	0.0118	0.45	-	-	[18]
Filtek Z350	13.45	0.17	0.00033	Bis-GMA, UDMA, TEGDMA, Dimethacrylate Filler content in 78.5 wt.% (Silica, zirconia, aggregated zirconia/silica)	[15]
Filtek Bulk Fill	13.46	0.18	0.00025	AUDMA, AFM, UDMA, DDDMA, EDMAB Filler content in 76.5 wt.% (Silica, zirconia, ytterbium trifluoride, aggregated zirconia/silica)	[15]
Polyurethane	3.60	0.30	-	-	[10]

**Table 3:** Stress peaks (MPa) during polymerization shrinkage measured in enamel and dentin tissue and the respective locations in the cavities.

Cavity Model and Restorative Material	Enamel Stress Peak (MPa)	Location	Dentin Stress Peak (MPa)	Location
Mesio-Occlusal Bulk-fill flowable	11.81	Cavo-surface angle	2.19	Lingual wall of the proximal box
Mesio-Occlusal Conventional	15.34	Cavo-surface angle	3.80	Lingual wall of the proximal box
Direct Access Bulk-fill flowable	10.09	Cavo-surface angle	3.44	Vestibular wall of the cavity
Direct Access Conventional	11.13	Cavo-surface angle	4.0	Vestibular wall of the cavity
Vertical Slot Bulk-fill flowable	12.99	Lingual wall of the proximal box	2.6	Dihedral linguogingival
Vertical Slot Conventional	17.03	Lingual wall of the proximal box	3.5	Dihedral linguogingival
Horizontal Slot Bulk-fill flowable	12.31	Cavo-surface angle	1.15	Vestibular wall of the cavity
Horizontal Slot Conventional	13.17	Cavo-surface angle	1.80	Vestibular wall of the cavity
Tunnel Bulk-fill flowable	13.10	Cavo-surface angle	3.8	Pulpal wall of the tunnel cavity

**Table 4:** Stress peaks (MPa) during polymerization shrinkage and loading effect measured in enamel and dentin tissue and the respective locations in the cavities.

Cavity Model and Restorative Material	Enamel Stress Peak (MPa)	Location	Dentin Stress Peak (MPa)	Location
Mesio-Occlusal Bulk-fill flowable	53.1	Cavo-surface angle	3.3	Lingual wall of the proximal box
Mesio-Occlusal Conventional	55.35	Cavo-surface angle	3.9	Lingual wall of the proximal box
Direct Access Bulk-fill flowable	36.12	Cavo-surface angle	3.12	Vestibular wall of the cavity
Direct Access Conventional	36.14	Cavo-surface angle	4.34	Vestibular wall of the cavity
Vertical Slot Bulk-fill flowable	56.08	Lingual wall of the proximal box	2.8	Dihedral linguogingival
Vertical Slot Conventional	56.14	Lingual wall of the proximal box	3.72	Dihedral linguogingival
Horizontal Slot Bulk-fill flowable	46.02	Cavo-surface angle	1.53	Vestibular wall of the cavity
Horizontal Slot Conventional	46.10	Cavo-surface angle	2.01	Vestibular wall of the cavity
Tunnel Bulk-fill flowable	46.71	Cavo-surface angle	4.01	Pulpal wall of the tunnel cavity

## Data Availability

Data available on request.
